# Molecular dynamics simulations of copper binding to amyloid-β Glu22 mutants

**DOI:** 10.1016/j.heliyon.2019.e03071

**Published:** 2019-12-31

**Authors:** Shaun T. Mutter, Matthew Turner, Robert J. Deeth, James A. Platts

**Affiliations:** a School of Chemistry, Cardiff University, Park Place, Cardiff, CF10 3AT, UK; b Department of Chemistry, University of Warwick, Gibbet Hill, Coventry, CV4 7AL, UK

**Keywords:** Theoretical chemistry, Molecular dynamics, Copper, Salt bridges, Amyloid peptide

## Abstract

We report microsecond timescale ligand field molecular dynamics simulations of the copper complexes of three known mutants of the amyloid-β peptide, E22G, E22Q and E22K, alongside the naturally occurring sequence. We find that all three mutants lead to formation of less compact structures than the wild-type: E22Q is the most similar to the native peptide, while E22G and especially E22K are markedly different in size, shape and stability. Turn and coil structures dominate all structures studied but subtle differences in helical and β-sheet distribution are noted, especially in the C-terminal region. The origin of these changes is traced to disruption of key salt bridges: in particular, the Asp23-Lys28 bridge that is prevalent in the wild-type is absent in E22G and E22K, while Lys22 in the latter mutant forms a strong association with Asp23. We surmise that the drastically different pattern of salt bridges in the mutants lead to adoption of a different structural ensemble of the peptide backbone, and speculate that this might affect the ability of the mutant peptides to aggregate in the same manner as known for the wild-type.

## 1 Introduction

Alzheimer's disease (AD) is characterised by the deposition of abnormal structures in the brain, particularly plaques – consisting of the Amyloid-β (Aβ) peptide – and neurofibrillary tangles. Aβ has two common isoforms, 40 and 42 residues in length, and is generated by sequential cleavage of the amyloid precursor protein (APP) by β- and γ-secretases. There are around fifteen known mutations of Aβ that may affect its structure and properties, and hence neurobiology. Formation of fibrils, probed by ThT fluorescence assays, was thought to be the key event in AD [1, 2], but more recent evidence suggests that small soluble Aβ oligomers are the key toxic species in the disease [3, 4]. Interestingly, more clinically severe mutations are associated with less ThT-responsive features [5]. In addition, Aβ variations at positions Ala21-Asp23 produce less ThT response over time than wild-type Aβ, despite forming aggregates [6]. Indeed, these mutants possess high aggregation rates [7], in agreement with the idea that non-ThT-responsive structures are involved in the AD process, while those that provide a ThT response are not necessarily pathogenic [8, 9, 10]. This is supported by data from a series of Glu22 (E22) mutants, which display accelerated formation of Aβ intermediates, increased neurotoxicity, but reduced fibril formation [7, 11].

The role of metal ions in AD is increasingly recognised, as disease progression correlates with the breakdown in homeostasis of copper, iron and zinc in the brain [12, 13, 14, 15, 16]. These ions play a key role in both the formation of aggregates and their neurotoxicity; concentrations of Cu(II) and Zn(II) are elevated in plaques of AD brains [17, 18], while plaques without these metals have been found to be non-toxic [19]. Furthermore, the redox activity of Cu(II) in particular provides a mechanism for damage to brain tissue via generation of reactive oxygen species (ROS) [20, 21]. The exact role and nature of these metal ions in AD is a subject of growing research interest, and has been extensively reviewed elsewhere [12, 13, 22, 23, 24]. Metal ion coordination has important effects on the structure and properties of Aβ, including aggregation propensity, though the recorded effects are diverse [13, 25]. In general, metal ions induce Aβ aggregation [26, 27, 28] though the type and toxicity of aggregate formed varies [29, 30, 31].

Cu(II) possesses high affinity towards Aβ [12, 32, 33] and dominates its coordination chemistry. A range of experimental and simulation studies have established details of Cu(II) coordination: the N-terminal region of the peptide contains the metal binding sites, though the exact nature of the coordinating residues depends on pH [34, 35, 36, 37, 38, 39]. Typically, Cu(II) binds through three N-donors and one O-donor, *via* Asp1/Ala2, His6 and His13/14, at physiological pH. Cu(II) may inhibit fibril formation, instead forming non-fibrillar aggregates and converting β-strand peptide structure into helices [39, 40]. The aetiology of disease onset is complex and not fully understood, but relative concentrations of metal and peptide can induce changes in the size and shape of aggregates formed [29, 32].

To date there have been very few studies of the effect of metal coordination on the structure, interactions or chemistry of Aβ mutants. In this work, molecular dynamics simulations were carried out on Cu(II) complexes with three E22 mutants, namely E22G, E22Q, and E22K, and compared to previous studies of the wild-type (WT) [41]. All are known mutants with established effects on aggregation and neurotoxicity. Moreover, they span a range of physico-chemical properties, from the anionic side chain in WT, through a polar but uncharged residue (E22Q) and small, uncharged amino acids (E22G), to a positively charged residue (E22K).

### 1.1 Computational methods

Wildtype Aβ1-42 was constructed within MOE [42] and Cu(II) was coordinated in the [O_c_^A2^,N_ε_^H6^,N_δ_^H13^,N_ε_^H14^] binding mode. Mutations were made using MOE's inbuilt sequence editor to generate the three E22 mutants. Residue protonation states were assigned to those appropriate for physiological pH values. Low mode molecular dynamics [43] simulations were carried out in the DommiMOE extension [44] to MOE, utilising previously reported Cu(II) ligand field molecular mechanics (LFMM) parameters [45] and AMBER PARM94 [46] parameters for all other atoms, to generate a diverse library of starting structures for further simulations. In particular, a combination of LFMM parameters from Type I copper protein with Cu–N bonding terms optimised for model Cu/imidazole/formamide complexes successfully reproduces DFT structures. Partial charge assignment was carried out using MOE's dictionary lookup feature and then copper and coordination sphere charges modified as reported previously [45]. We note that other binding modes are known, but our goal here is to compare mutants with a common coordination to copper, not to explore all available binding sites. The functional form of the LFMM implementation of AMBER, in which M—L bonds are described with a Morse potential, means that metal-ligand dissociation is effectively impossible, at least at the temperatures and over the timescales used here.

Ligand field molecular dynamics (LFMD) simulations were carried out using the DL_POLY_LF code [47], which incorporates LFMM within the DL_POLY_2.0 package [48]. All simulations were carried out using an NVT ensemble, with a Nose-Hoover thermostat with relaxation constant of 0.5 ps, at a temperature of 310 K. Implicit solvation was modelled through the reaction field model with dielectric suitable for bulk water (ε = 80) with cutoffs of 10 and 21 Å, for van der Waals interactions and long range electrostatics, respectively. Use of implicit solvent has been shown to enhance conformational sampling of flexible systems [49]. All bonds to hydrogen were constrained using the SHAKE algorithm [50], with 10^−8^ Å tolerance. All simulations were run for 1 μs, with a 1 fs integration timestep used throughout. Atomic positions were recorded every 10 ps for trajectory analysis.

All analysis of LFMD trajectories was carried out using VMD 1.9.2 [51]. Root mean square deviation (RMSD) and radius of gyration (Rg) were used as indicators of equilibration. The VMD timeline extension was used for secondary structure, root mean square fluctuations (RMSF), salt bridge, and hydrogen bond analysis. Tertiary structure Cα contact maps were created using the ITrajComp plugin [52]. Hydrogen bond presence was determined by a distance of less than 3 Å and angle of less than 20° between donor and acceptor. Salt bridge presence was determined by less than 3.2 Å between O and N atoms on charged residues: this definition means that it is possible for a residue to form multiple simultaneous salt bridges, so the total percentages for any given residue may exceed 100%.

## 2 Results and discussion

**Figure 1:**
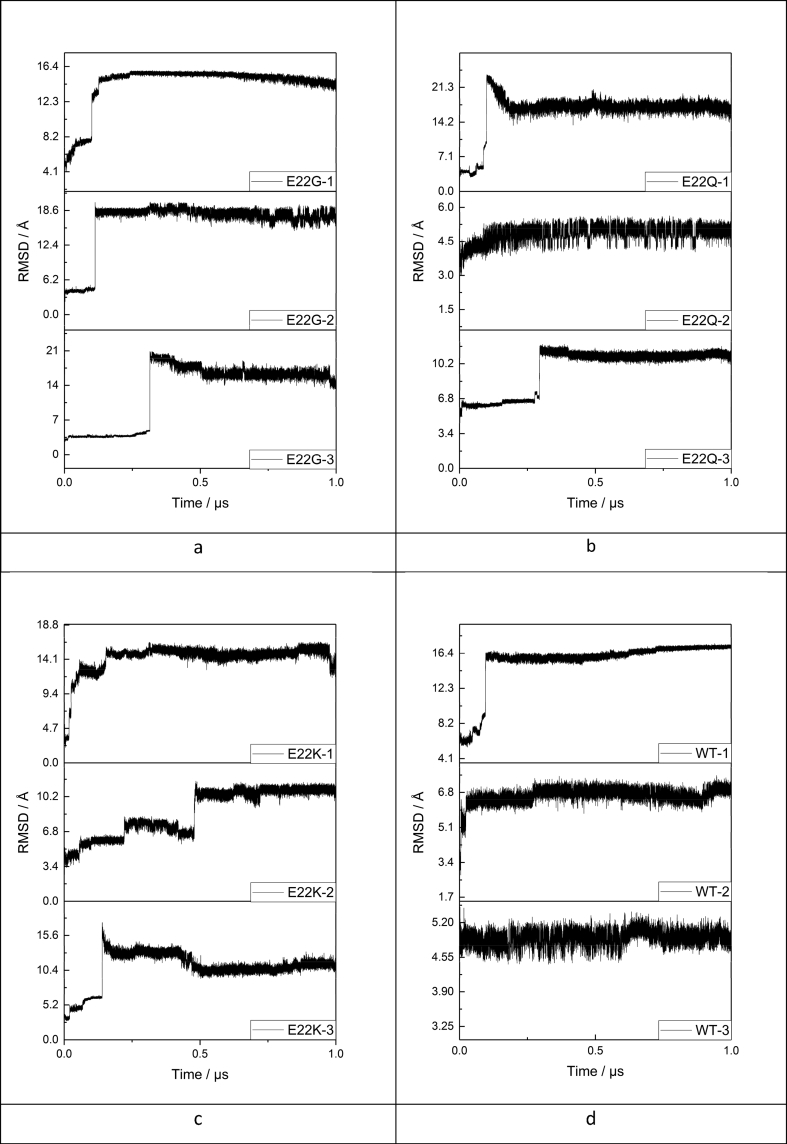
RMSD (Å) from initial structure of each trajectory, against time (μs): a) E22G, b) E22Q, c) E22K and d) WT.

Three low energy structures generated by low mode molecular dynamics, with mutual RMSD greater than 1.5 Å, were chosen as separate starting points for LFMD simulations, to allow for more effective sampling of conformational phase space. Microsecond LFMD simulations were carried out for each of the three starting points, for each mutant, and associated RMSD plots are reported in Figure 1. The intrinsically disordered Aβ peptide offers complications when equilibrating MD simulations. As such, full equilibration would only occur on timescales beyond current computational capabilities. Therefore we have utilised the description of quasi-equilibration, as reported by Huy *et al.* [53], where RMSD fluctuation around a stable point is sufficient to consider a simulation equilibrated.

For our systems, timescales in the order of hundreds of nanoseconds are required: quasi-equilibration for E22G required 200, 200, and 500 ns of simulation; E22Q required 250, 250, and 300 ns; E22K required 300, 500, and 500 ns; while WT required 200, 300, and 100 ns for runs 1–3 respectively. Table 1 reports statistics drawn from RMSD values for the quasi-equilibrated portion of each trajectory. All three simulations for all three mutants result in low standard deviation values, showing that beyond the equilibration point the trajectories are generally stable.

**Table 1:** RMSD (Å) of E22 mutants.

	avg RMSD	SD	max	min
E22G-1	2.28	0.57	0.42	5.29
E22G-2	4.73	1.08	1.11	6.72
E22G-3	5.41	1.13	1.14	8.48
E22Q-1	4.89	0.70	1.34	8.12
E22Q-2	1.22	0.19	0.60	2.15
E22Q-3	2.44	0.35	0.81	4.21
E22K-1	5.88	0.44	0.95	7.29
E22K-2	2.77	0.66	0.79	5.07
E22K-3	3.89	0.60	1.27	6.70
WT-1	16.39	0.58	17.56	14.98
WT-2	6.77	0.23	7.93	5.78
WT-3	4.92	0.13	5.40	4.42

Rg values for individual trajectories show stable values past the equilibration times noted above: Table 2 reports values averaged over all post-equilibration trajectories. High standard deviation values are a result of the combination of multiple trajectories: variation is much smaller within trajectories. WT has the lowest average Rg, indicating the most compact structure: the mean value compares well with literature [54]. Ref 54 reports Rg of Aβ1-42 in the range 9–13 Å, with a mean of 1.14 nm, while values of 10–15 Å are quoted in ref [55]. E22Q is only slightly larger on average, with increased Rg of around 2 Å, respectively. However, mutation to the small, achiral glycine (E22G) or the positively charged lysine (E22K) result in the most obvious differences in compactness of structure, with average values increased by almost 8 and over 5 Å, respectively.

**Table 2:** Radius of gyration (Å) of E22 mutants.

	avg R_g_	SD	max	min
E22G	21.59	1.46	24.28	15.88
E22Q	15.81	3.75	23.68	10.59
E22K	18.95	3.02	23.73	14.08
WT	13.78	5.53	22.83	8.82

Data relating to the hydrogen bonding within mutants is reported in Table 3. As with our previous study on WT, hydrogen bonds in all mutants considered are highly transient. High standard deviation values relative to the average number of H-bonds, along with minimum numbers as low as zero and maximum numbers as high as 25, are indicative of transience. Common H-bonds, reported as donor-acceptor, include Asn27 backbone-Asp23 backbone (42% incidence) and Gln15 sidechain-Glu11 sidechain (38%) for WT; His14 backbone-Asp7 sidechain (31%) and Ser26 sidechain-Asp23 sidechain (24%) for E22G; Ser26 sidechain-Asp23 sidechain (44%) and His14 sidechain-Glu11 sidechain (38%) for E22Q; and Ser8 sidechain-Asp7 sidechain (40%) and Asn27 backbone-Ala42 backbone (21%) for E22K. Several H-bonds fitting the expected i+4 → i pattern for α-helices are observed, including N27-D23 in WT, consistent with secondary structure patterns discussed below.

**Table 3:** Hydrogen bond count for E22 mutants.

	avg H-bond #	SD	max	min
E22G	8.46	2.26	18	0
E22Q	10.31	2.58	22	1
E22K	8.80	2.86	22	0
WT	9.90	2.63	25	1

**Figure 2:**
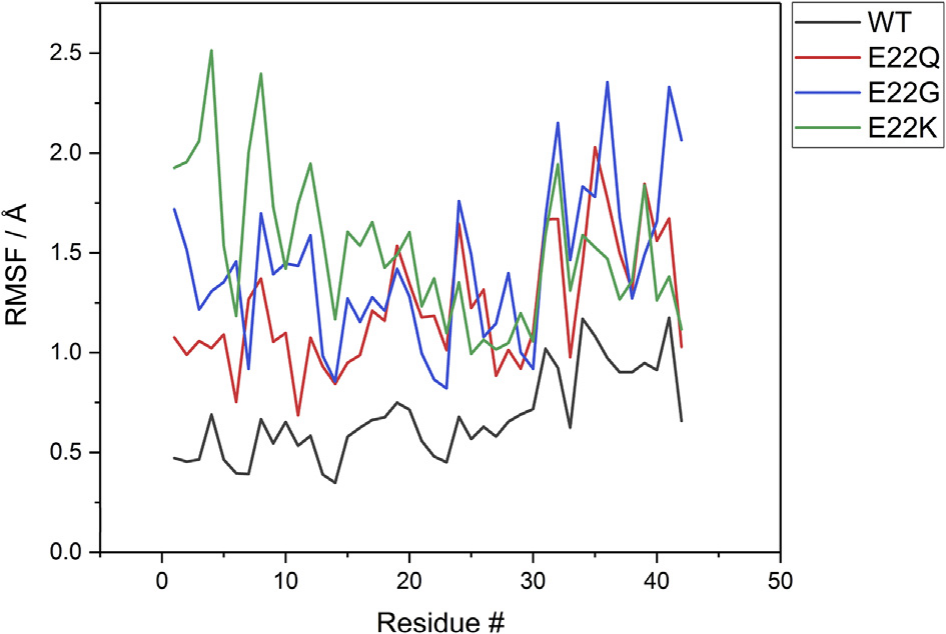
Root mean square fluctuation (Å) of E22 mutants.

Figure 2 shows the RMSF of the mutants by residue, compared to the wildtype. WT exhibits the lowest RMSF for all residues compared to the mutated peptides. Interestingly, the mutated residues are not necessarily those with largest RMSF values; this is somewhat surprising due to the different chemical nature of the residues involved. Figure 2 indicating that the effect of mutation on peptide flexibility is highly non-local. In general, the C-terminus exhibits larger RMSF values than the N-terminus, as expected due to the anchoring effect of coordination of Cu(II) to three N-terminal residues. However, E22K displays a different pattern: the N-terminus has larger RMSF values than the C-terminus, with the coordinating residues having relatively low values but many of the others in the metal binding region exhibiting high mobility, notably Asp1-Phe4, Asp7-Ser8 and Val12.

**Figure 3:**
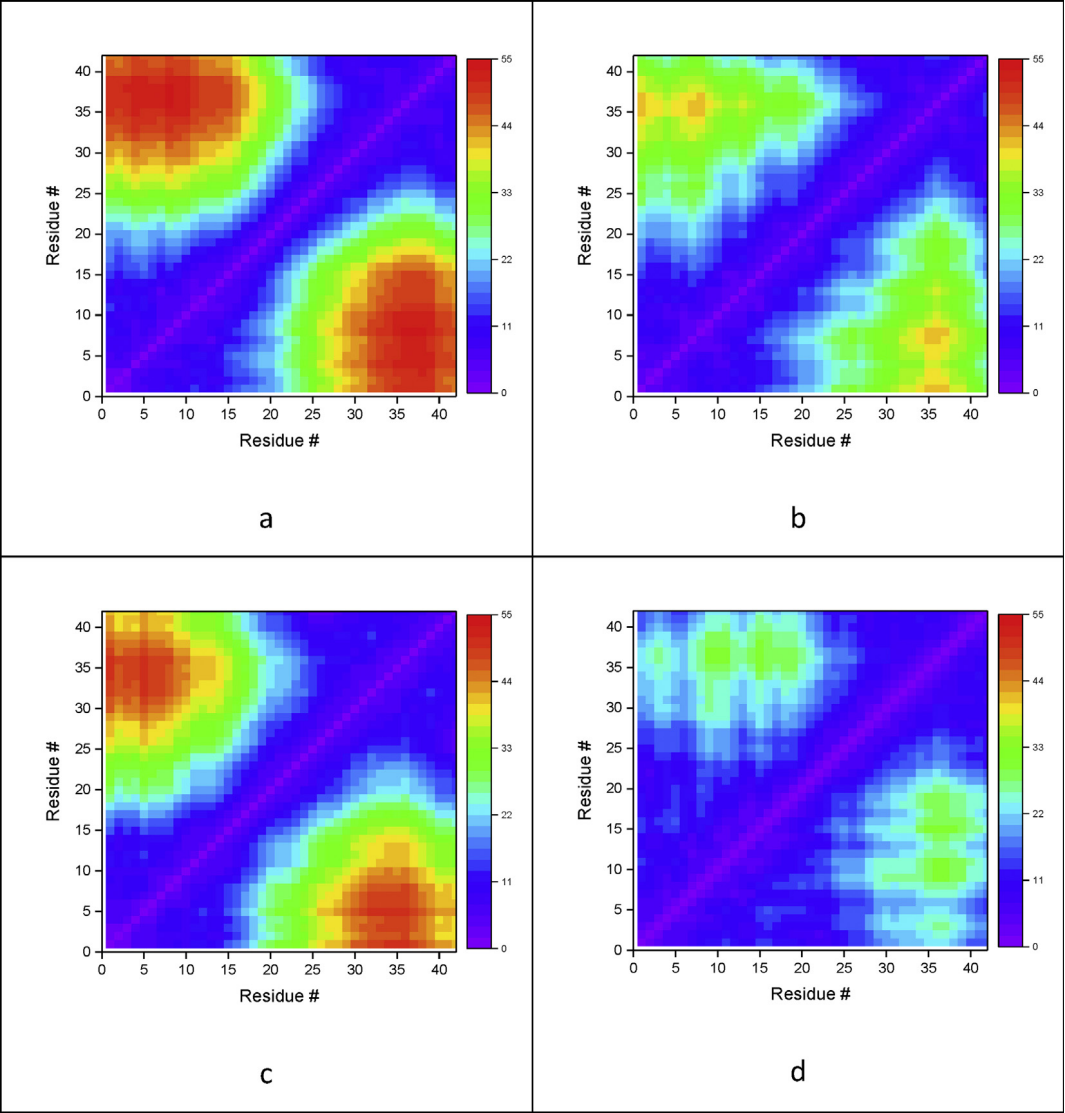
Residue contact maps, based on Cα distances (Å): a) E22G, b) E22Q, c) E22K, d) WT.

**Figure 4:**
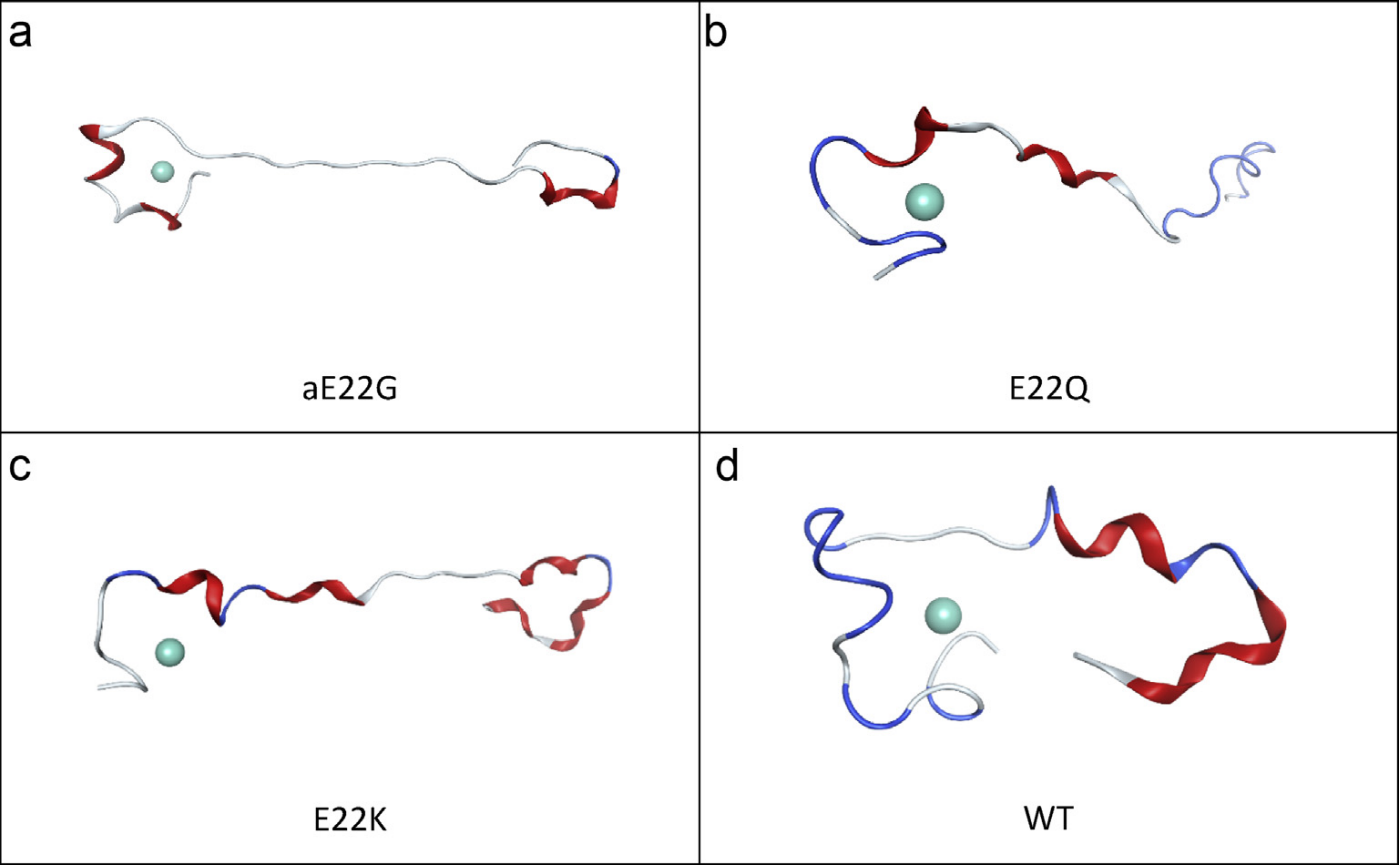
Final frames of LFMD trajectories for a) E22G, b) E22Q, c) E22K, d) WT.

Contact maps are a useful measure of the average shapes of dynamical systems and have been utilised to compare the different mutants here. Figure 3 reports contact maps between the α-carbons of each residue for the mutants and the wildtype. WT has a relatively compact structure, with longest Cα-Cα distances of *ca.* 30 Å between Ser8-Phe20 and Lys28-Val40. E22Q shows a more extended structure: distances of *ca*. 40 Å for N-terminal residues (up to Gly9) with C-terminal residues Ile32-Gly38. Mutation to the oppositely charged lysine (E22K) results in a strikingly different contact map, with much greater separation between the termini, corresponding to an extended structure. This is observed to an even greater extent in the E22G mutation, wherein Cα-Cα contacts between the termini exceed 50 Å for residues up to Gln15 with Il32-Val40. Structures of final the final frames of MD trajectories are also reported in Figure 4, which are in agreement with the findings for the contact maps.

**Figure 5:**
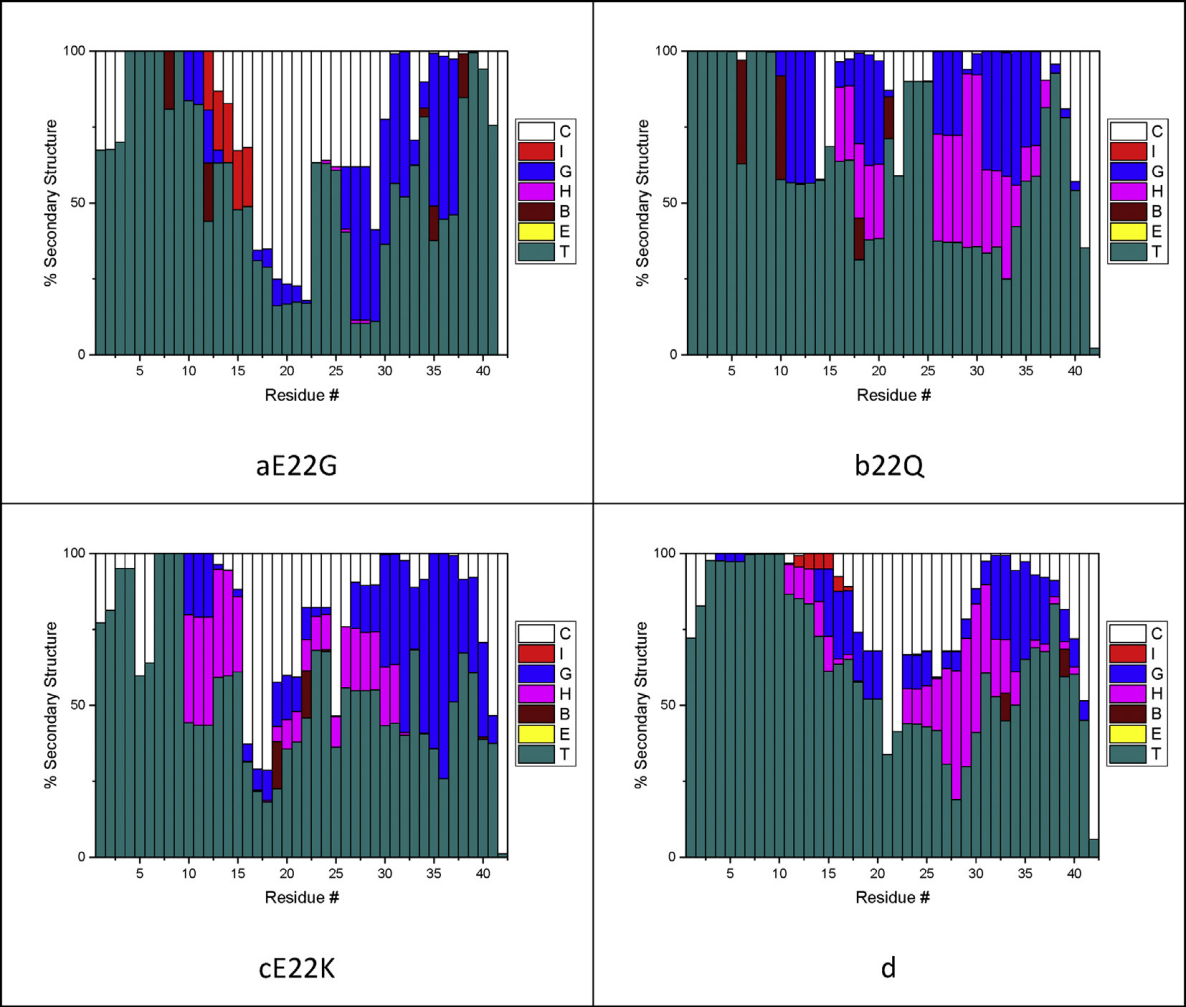
Percentage secondary structure of E22 mutants. C, I, G, H, B, E, and T, correspond to the structure elements coil, π-helix, 3_10_ helix, α-helix, β-bridge, β-sheet, and turn, respectively: a) E22G, b) E22Q, c) E22K, d) WT.

Secondary structure analysis was carried out using the STRIDE algorithm: percentage secondary structure against residue number is reported in Figure 5. A breakdown of the overall contributions of secondary structure elements for each mutation is also reported in Table 4. As expected for intrinsically disordered peptides, the major constituents of the secondary structure profile are turn and coil. These structural elements correspond to a lack of order and comprise over 70% of the total peptide structure for all systems. Interestingly, there is considerable variation in helix and sheet content between the mutants. All systems have very little β-sheet character: the largest being 2.3% for E22Q, found in the in the metal binding region as well as the peptide's central hydrophobic core (Leu17-Ala21). In contrast, the WT peptide adopts sheet-like conformations exclusively at the C-terminus.

**Table 4:** Breakdown of percentage secondary structure for E22 mutants.

	Helix %	Sheet %	Turn/Coil %
E22G	15.4	1.6	83.1
E22Q	25.2	2.3	72.5
E22K	25.3	0.8	73.9
WT	18.6	0.4	81.0

There are also differences in helical content across the mutants, 25% in E22K compared to 19% in WT. These consist of a mix of π, 3_10_ and, α-helices: the latter two making up the majority. These are primarily seen in the regions of Tyr10-Gln15 and Gly25-Val40 residues. WT and E22G, but not E22Q nor E22K, exhibit π helices in the metal binding region site near His13 and His14. Some π-helical character is also observed toward the C terminus in some mutants but at much lower occurrences. A distinction can also be made between turn and coil structures: the mutants that result in the most extended structures, E22G and E22K, have the greatest concentration of coil structure. This character is centred on the central hydrophobic region and toward the C terminus. The coil character therefore indicates this presence, whilst E22Q and WT indicates a greater propensity to remain globular.

**Figure 6:**
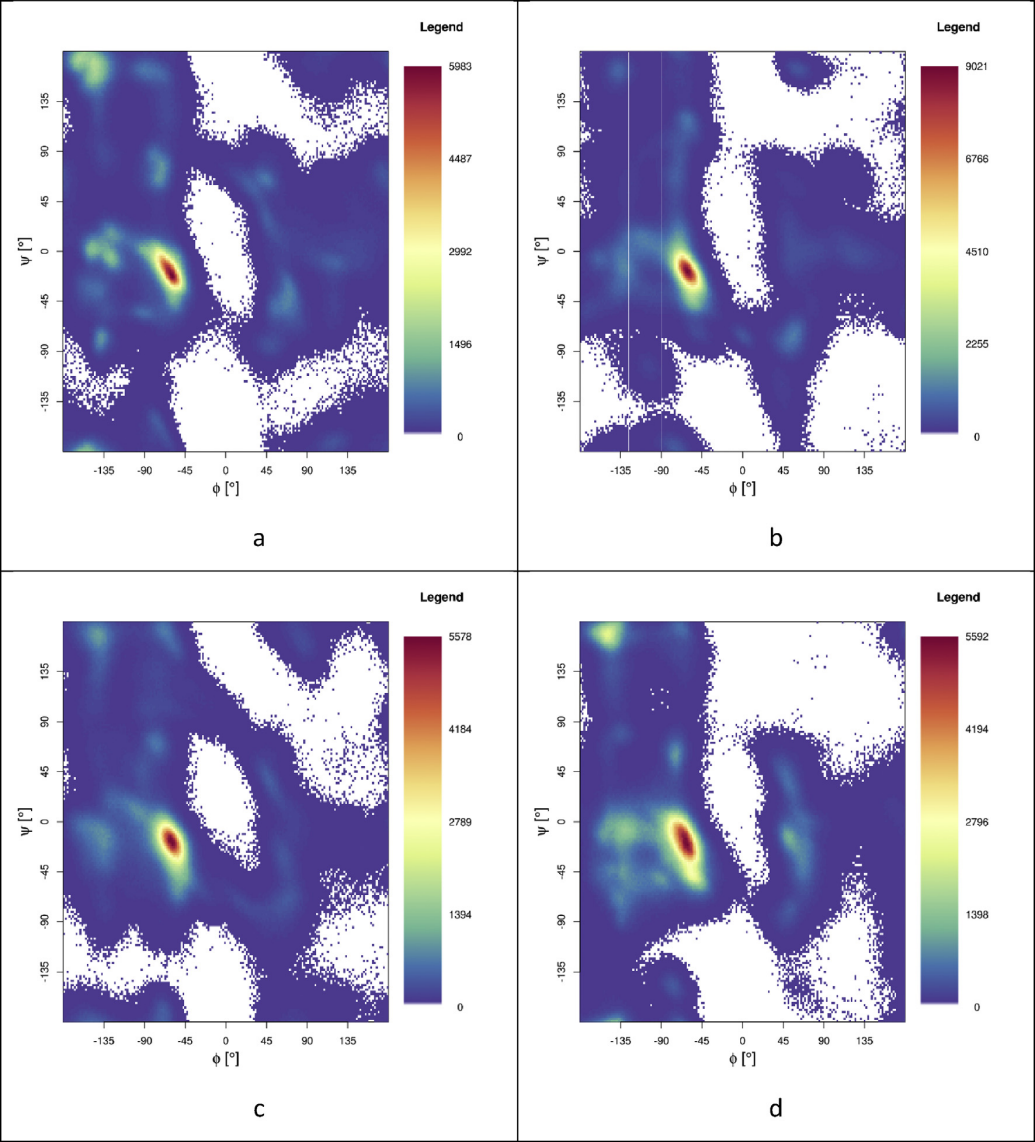
Ramachandran plots of a) E22G, b) E22Q, c) E22K, d) WT.

Ramachandran maps (Figure 6) shed further light on secondary structure: for WT Aβ, most conformations adopt right-handed helical-like conformations, centred around (-60, -20). Interestingly, there are many further conformations located around (-135, -15), close to the helical region of the plot. In addition, there are notable contributions from left-handed helical structures at (45, -15) and β-sheet type structures at (-160, 160). E22K and E22Q mutants exhibit similar Ramachandran maps, dominated by right-handed helical-like conformations, indicating that these mutations have relatively little effect on the total backbone conformations sampled. This reflects their similar secondary structure profiles. E22Q reports the highest incidence of β-sheet structure, but has relatively few conformations in this region of the plot, indicating that while mutants adopt sheet-like conformations, they lack the requisite hydrogen bonds to be classified as β-sheets. E22G is also dominated by helical-type conformations, but also contains more β-sheet structures. This is in agreement with other data illustrated here; this mutation exhibits the second-highest degree of β-sheet structure, as well as the most extended conformation.

**Figure 7:**
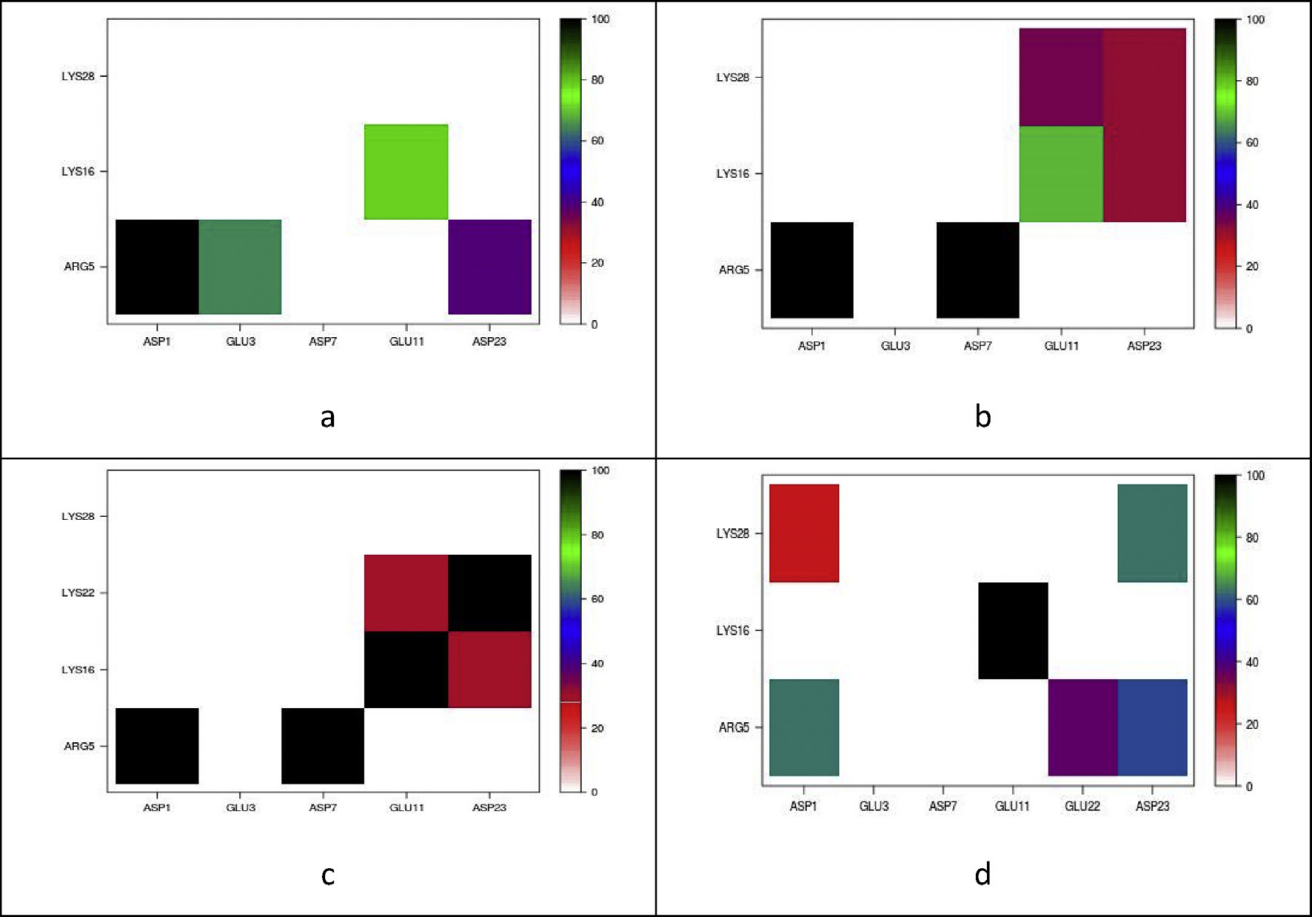
Percentage occurrence of salt bridges for a) E22G, b) E22Q, c) E22K, d) WT.

Salt bridge interactions strongly influence peptide structure and stability. The natural peptide has nine charged residues at physiological pH, three positive and six negative, resulting in a possible eighteen salt bridges: E22Q and E22G have fifteen possible bridges, and E22K twenty. Salt bridge contact maps for each structure are reported in Figure 7. All systems show similarities in the metal binding region, which may be expected due to their identical copper binding modes. The Asp1-Arg5 salt bridge is present at close to 100% of the time for all mutants, but just 63% for WT. WT contains an Asp1-Lys28 salt bridge (27%), which is not present in the mutants, reflecting the more compact structure of the WT compared to the mutants (*vide supra*). Other differences in this region include the presence of Glu3-Arg5 interactions in E22G, which are not observed for the other systems. Lys22 in E22K forms new salt bridge interactions, particularly with Glu11 (29%) and Asp23 (100%): these new interactions are formed at the expense of those with Lys28, observed in other mutants. Reduction of the stabilising interactions of Lys28 with the closer Lys22 therefore seems to be the likely origin of the extended conformation observed from the contact maps above.

**Figure 8:**
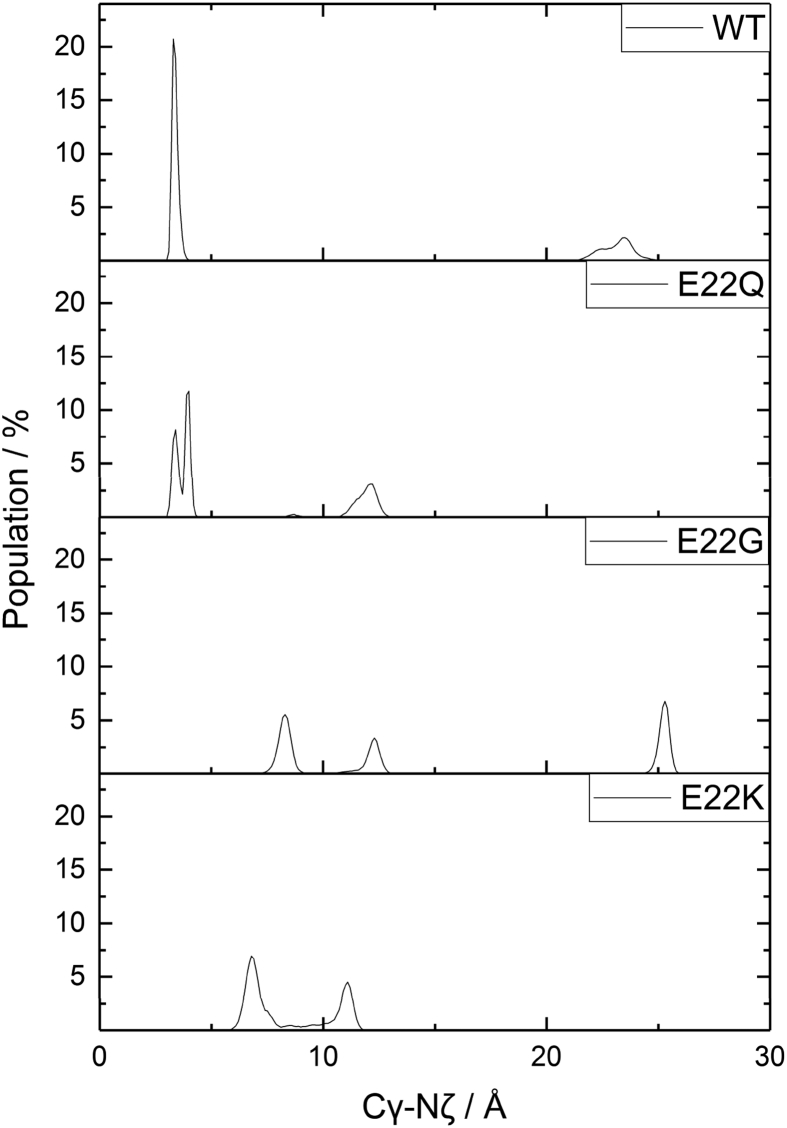
Asp23-Lys28 salt bridge distribution (Å) against percent of occurrence.

The Asp23-Lys28 bridge plays an important role in the aggregation behaviour of Aβ [56]: mutation of a residue directly adjacent seems likely to have an impact. To examine this influence, the Asp23-Lys28 distance has been plotted for the mutants and WT in Figure 8. WT exhibits a sharp peak at 3.5 Å, and a much shallower, broad peak above 20 Å, illustrating the presence of two conformations. A similar profile is observed for E22Q, with the same sharp peak at 3.5 Å and smaller, broader peaks at longer distances (12–14 Å). The two mutations that have no Asp23-Lys28 salt bridge interactions have no peaks below 5 Å E22G, the most extended system, has a peak at *ca.* 25 Å, as well as two at 8 and 13 Å, while E22K lacks the peak at very long distance but exhibits peaks around 8, 15, and 17 Å.

## 3 Conclusions

We report ligand field molecular dynamics simulations of the Cu(II) complexes formed by three different Glu22 mutants of the amyloid-β 1–42 peptide: namely E22G, E22Q and E22K. All are known to increase the rate of peptide aggregation and the likelihood of developing the symptoms of Alzheimer's disease. Three independent simulations of one microsecond for each system were performed, each reaching pseudo-equilibration after several hundred nanoseconds. Analysis of frames collected after equilibration indicates major differences between mutants and the wild-type peptide. E22Q is the most similar to the native peptide, but even here subtle differences are evident. E22G and especially E22K are markedly different in size, shape and stability, both adopting much more extended structures that are much more flexible. Somewhat surprisingly, changes induced by mutations are apparent across the entire peptide: root mean square fluctuation in particular shows that E22K induces major changes in the N-terminal sequence, up to 20 residues away from the site of mutation, while E22G causes the C-terminus to become much more flexible. In common with a previous MD study of mutated Aβ [57], turn and coil structures dominate all structures studied but subtle differences in helical and β-sheet distribution are noted, especially in the C-terminal region. All mutants, as well as WT, sample a wide set of structural ensembles: this structural diversity and the conformational may facilitate the interconversions between various secondary and tertiary structures that accompany aggregation of Aβ.

The origin of these difference is apparently disruption to the salt-bridge network: E22Q has a strongly populated Arg5-Asp7 interaction that is absent in WT, while the Glu11-Lys16 bridge that is frequently populated in WT is much reduced. E22K leads to a quite different pattern of salt bridges, with the mutated residue itself forming interactions with Glu11 and especially Asp23. E22G leads to complete loss of the Asp1-Lys28 interaction and diminution of Glu11-Lys18. Both mutations therefore leads to substantial reduction in the interactions that keep the wild-type peptide in a relatively compact conformation, and hence to the extended conformations noted above. While we cannot draw direct conclusions on the effect of mutation on aggregation from these simulation of monomers, it is intriguing that the E22G and E22K are known to give rise to “small protofibrils and oligomers” and to “less fibrillar” aggregates, respectively [58]. We speculate that the loss of salt bridges within the monomer and the resulting extended structure give rise to different aggregation behaviour, and that the characteristic fold of Aβ seen in mature fibrils may be less favourable in the absence of key salt bridges such as Asp23-Lys28.

It is appropriate at this stage to discuss limitations of this work. Firstly, we have only studied 1:1 Cu:peptide complexes, and then only in one of several possible coordination modes. This may not be representative of the more complex *in vivo* situation, but serves as a basis for comparison of mutants without further complication of changing stoichiometry or coordination. Secondly, we have also only simulated monomeric Aβ whereas oligomers are thought to be the key species in disease onset: we hope to report simulations of larger systems in future publications, but at present we can only infer potential interactions from the properties of the monomer. Thirdly, use of an implicit solvent model prevents the simulations from accounting for any explicit role of water molecules in metal coordination. Despite these limitations, we have identified important differences in the structure and dynamics of the mutated peptides and their interaction with Cu(II) that give some insight into how they behave in practice.

## Data Statement

Frames taken from all trajectories have been deposited in PDB format, available from https://doi.org/10.5281/zenodo.2537978.

## Declarations

### Author contribution statement

Jamie Platts: Conceived and designed the experiments; Analyzed and interpreted the data; Contributed reagents, materials, analysis tools or data; Wrote the paper.

Shaun Mutter: Conceived and designed the experiments; Performed the experiments; Analyzed and interpreted the data; Wrote the paper.

Matthew Turner: Analyzed and interpreted the data; Contributed reagents, materials, analysis tools or data.

Rob Deeth: Contributed reagents, materials, analysis tools or data.

### Funding statement

This work was supported by EPSRC under grant reference EP/N016858/1. The authors are grateful to Cardiff University's ARCCA for computing resources.

### Competing interest statement

The authors declare no conflict of interest.

### Additional information

No additional information is available for this paper.
